# From Power‐Law to Correlation‐Time Distributions: A Unified Framework for the Analysis of Nuclear Magnetic Relaxation Dispersion (NMRD) Profiles of Complex Biological Systems

**DOI:** 10.1002/mrc.70093

**Published:** 2026-03-05

**Authors:** Giacomo Parigi, Adam Kubrak

**Affiliations:** ^1^ Magnetic Resonance Center (CERM) University of Florence Sesto Fiorentino Italy; ^2^ Department of Chemistry “Ugo Schiff” University of Florence Sesto Fiorentino Italy; ^3^ Consorzio Interuniversitario Risonanze Magnetiche Metallo Proteine (CIRMMP) Sesto Fiorentino Italy

**Keywords:** correlation times, field cycling, model free, NMR, NMRD, power law, relaxation dispersion, relaxometry

## Abstract

A recurring and significant finding across diverse biological and macromolecular systems is that the frequency dependence of the spin–lattice relaxation rate often cannot be well fitted with a single correlation time but rather follows a power‐law function. This power‐law dependence is attributed to the dynamics of rare, strongly bound water molecules trapped on rugged macromolecular surfaces, with a Pareto distribution of correlation times. Here, we show that power‐law dependences naturally emerge from a broad distribution of correlation times with weighting factors proportional to 1/*τ* ^(1−*α*)^. We derive analytical expressions for limiting cases and perform numerical simulations demonstrating that this distribution of correlation times generates power‐law exponents closely matching *α* over wide frequency windows. We validate this framework by fitting Nuclear Magnetic Relaxation Dispersion (NMRD) profiles of sedimented proteins, biological tissues, cross‐linked hydrogels, and protein solutions. This approach establishes a physical interpretation of power‐law relaxation, enabling the extraction of dynamic information otherwise inaccessible.

## Graphical abstract

^1^H NMRD profiles of biological systems often follow a power‐law dependence rather than a Lorentzian dispersion. This behavior can arise from a broad distribution of correlation times, weighted by 1/τ ^1−*α*^. Validated across diverse systems such as sedimented proteins and biological tissues, this framework provides a physical interpretation of power‐law relaxation and enables direct access to dynamic information.

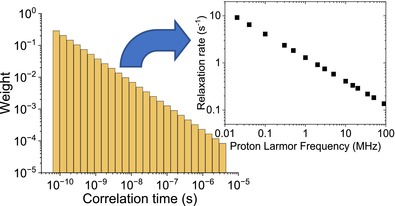

## 1 Introduction

Power‐law functions are increasingly used in the analysis of Nuclear Magnetic Relaxation Dispersion (NMRD) profiles as the spin–lattice relaxation rates often exhibit a power‐law dependence on the resonance frequency, which translates in a linear dependence in Log–Log plots. This behavior is especially common in the presence of complex molecular dynamics in heterogeneous systems [1].

NMRD profiles report the nuclear longitudinal relaxation rates, *R*_1_, as a function of the proton Larmor frequency over a quite broad range typically spanning from 10 kHz to 40 MHz. This wide frequency window permits investigating dynamic processes on timescales from nanoseconds to microseconds and beyond from the position of the relaxation dispersion. It is worth mentioning that this time window is out of range for conventional high‐field NMR, which usually probes only motions in the sub‐nanosecond time scale [2, 3]. In fact, the frequency dependence of the relaxation rates is determined by the time constants describing the fluctuations in the energy of the time‐dependent interactions between the observed nuclei and their surroundings, called correlation times. Furthermore, the shape of the NMRD profiles is a fingerprint of the underlying motional mechanism, such as three‐ or two‐dimensional translational diffusion, molecular tumbling, internal mobility, Rouse, or reptation dynamics of polymeric unentangled or entangled chains.

For complex systems containing macromolecular components, such as lowly hydrated proteins, biopolymers, hydrogels, and biological tissues, the observed dependence of relaxation on the nuclear Larmor frequency frequently deviates from the simple Lorentzian spectral density function, which is typical of relaxation processes due to rigid molecular tumbling in liquid solutions. In the low‐frequency range, these systems often show a sharp increase in relaxation as frequency decreases. This behavior is expected to originate from slow molecular motions, often attributed to rare water molecules trapped in potential energy wells on the macromolecular surfaces, and thus experiencing long residence times. To accurately model this complex, non‐Lorentzian behavior, the data are often analyzed using power‐law functions. In this phenomenological approach, the relaxation rate is expressed as an inverse power of the applied frequency *R*_1_ = *Cω* ^−*β*^, where *β* is the power‐law exponent, determined from the fit of the data. The exponent *β* is in many cases linked to the physical mechanism of the molecular motion and to the degree of restriction experienced by the observed nuclei [1, 4].

Due to the ability of power‐law models to reproduce the frequency dependence of relaxation rates of human tissues at low fields, they are increasingly used as diagnostic markers in biological and medical studies. Pathological alterations in tissues, in fact, often affect the slow dynamics within macromolecules and of water molecules strongly interacting with them. Endometrial cancer tissues, for instance, exhibit power‐law frequency dependences with exponent ranging from 0.31 to 0.42 [5], whereas lung tissues show exponents from 0.27 to 0.33 [6]. Similarly, in subacute ischemic stroke, the exponent measured in infarcted brain regions is significantly lower than in unaffected brain tissue (median 0.23 vs. 0.35) [7].

The origin of this power‐law dependence has been attributed to the dynamics of water molecules at the nonuniform, rugged macromolecule interfaces [4, 8, 9]. At these interfaces, in fact, the water molecules become highly trapped and undergo slow dynamics governed by rare transitions out of deep potential wells. An exponential distribution of energy barriers yields a Pareto distribution of the associated correlation times, which results in power‐law dependences. This distribution implies that a very small population of strongly trapped, long‐lived water molecules dictates the frequency dependence of relaxation at low fields, rather than the majority of the interfacial water molecules.

Previous theoretical work on lowly hydrated proteins and biopolymers predicts power‐law dependences with exponent characteristic of specific mechanisms of motion (e.g., Rouse or reptation dynamics and intramolecular or intermolecular dipole–dipole interactions). The exponent *β* typically ranges between 0.25 (in Rouse or reptation dynamics, where mobility is highly restricted) and 0.80 (for water molecules probing protein backbone dynamics via cross‐relaxation effects [4]). On the other hand, the NMRD profiles of water‐pectin mixtures required combining a term for two‐dimensional (surface) diffusion of water molecules with a power‐law term, attributed to the motion of the pectin chains and probed by bound water molecules, yielding exponent *β* values as low as 0.15–0.20, indicative of highly restricted long‐range mobility [10]. Similarly, the NMRD profiles of fish collagen gels [11] could be analyzed using either a complex model incorporating surface diffusion or a power law model to capture the full frequency dependence across all samples and temperatures.

A significant drawback of power law descriptions is the lack of characteristic timescales that can inform on the correlation times of the mobility processes responsible for relaxation. On the other hand, a power‐law relaxation profile can be reproduced by summing Lorentzian functions with a distribution of correlation times, as it can be obtained from an extended model‐free approach [12]. Recovering the distribution of correlation times and their corresponding weights from a power‐law profile is however an ill‐posed inverse problem, which admits an infinite number of solutions. A reliable analysis of the profile thus requires introducing a model describing the functional distribution of the correlation times (i.e., their weights as a function of the correlation time) [13, 14, 15]. We show here that the simple weight function 1/*τ* ^(1−*α*)^, with *α* between 0 and 1, and *τ* varying in a wide range of values between *τ*_min_ and *τ*_max_, indeed produces a power law dependence of the relaxation rates over a wide frequency range. Recovering this weight function thus makes it possible to evaluate the relative contributions of the different correlation times, providing insight into the characterization of the underlying dynamics processes.

## 2 Theoretical Background

Classical relaxation theories assume isotropic motion and exponentially decaying correlation functions. This assumption leads to spectral densities characterized by Lorentzian functions. In the simple case of proton–proton dipole–dipole interactions modulated by stochastic fluctuations occurring with a single correlation time $\tau_{c}$, the ^1^H longitudinal relaxation rates are given by [16]

(1) $$R_{1}=a+b\left[\frac{\tau_{c}}{1+\omega_{I}^{2}\tau_{c}^{2}}+\frac{4\tau_{c}}{1+4\omega_{I}^{2}\tau_{c}^{2}}\right]$$

where *ω*_*Ι*_ = −*γ*_*I*_ *B*_0_ is 2π times the proton Larmor frequency, *B*_0_ is the applied magnetic field, and γ_I_ is the proton magnetogyric ratio. The constant $b=f_{M}\frac{2}{5}{\left(\frac{\mu_{0}}{4\pi }\frac{\hbar \gamma_{I}^{2}}{r^{3}}\right)}^{2}I\left(I+1\right)$ is proportional to the squared interaction energy and to the molar fraction $f_{M}$ of protons with correlation time $\tau_{c}$ and in fast exchange. The constant *a* accounts for the high field relaxation rate determined by the contributions of all protons with much shorter correlation times.

Whereas in diluted water solutions of small molecules, the correlation time is usually of the order of tens to hundreds of picoseconds, in the presence of macromolecules, this correlation time largely increases. In fact, the dynamics of the water molecules, for instance, on the coordination surface of the macromolecules, or transiently trapped into their cavities, is slowed down by several orders of magnitude. Therefore, the proton dipole–dipole interactions are modulated with a correlation time given by the shortest between their lifetime onto the macromolecule surface and the reorientation time of the whole macromolecule, or, when internal (local) dynamics is present, the corresponding faster internal motional time [3].

When Equation (1), containing a single correlation time, fails to provide a satisfactory fit to the experimental profiles, as often occurs for complex and heterogeneous systems, an “extended” model‐free approach is typically employed, in which multiple correlation times are considered [2, 3, 17, 18]. This approach is justified because the numerous interactions between the many water molecules and the macromolecules can be modulated with a wide range of correlation times with a given weight. As a result, the water proton longitudinal relaxation rate can be empirically described as a sum of Lorentzian dispersions [2, 3, 17, 18, 19, 20],

(2) $$R_{1}=a+b\sum_{n}c_{n}\left[\frac{\tau_{n}}{1+\omega_{I}^{2}\tau_{n}^{2}}+\frac{4\tau_{n}}{1+4\omega_{I}^{2}\tau_{n}^{2}}\right]$$

where $c_{n}$ is the weight coefficients summing to 1 and $\tau_{n}$ is the *N* correlation times. Generally speaking, a good fit with Equation (2) can be easily achieved; however, it may be possible that no physical meaning can be attributed to the obtained correlation times.

Water molecules in highly heterogeneous environments can thus experience a distribution of correlation times, which may arise from restricted local reorientational motions or from jumps between distinct local environments. Assuming an exponential probability distribution for the activation energy (in which the probability decreases exponentially with the energy value), the resulting distribution of correlation times is weighted as 1/*τ* ^(1−*α*)^ (a form known as a Pareto distribution) [8, 9].

In the next sections, it will be shown that if a distribution of correlation times is chosen to be homogeneously spaced on a logarithmic scale between *τ*_min_ and *τ*_max_, with $c_{n}=\frac{\frac{1}{\tau_{n}^{1-\alpha }}}{\sum_{n}\frac{1}{\tau_{n}^{1-\alpha }}}$ (inverse‐scaling distribution), then the field dependence of the relaxation rate is well described by a power law over a wide frequency range between 1/*τ*_max_ and 1/*τ*_min_.

## 3 Results and Discussion

The relaxation rates obtained from a distribution of Lorentzian functions where the correlation times are weighted as 1/*τ* ^(1−*α*)^ and homogeneously spaced on a logarithmic scale over the range from *τ*_min_ to *τ*_max_ can be calculated as follows:

(3) $$R_{1}=a+b\frac{\int_{\tau_{\min}}^{\tau_{\max}}\frac{1}{\tau^{1-\alpha }}\left[\frac{\tau }{1+\omega_{I}^{2}\tau^{2}}+\frac{4\tau }{1+4\omega_{I}^{2}\tau^{2}}\right]\;\mathrm{dLo}g_{10}\left(\tau \right)}{\int_{\tau_{\min}}^{\tau_{\max}}\frac{1}{\tau^{1-\alpha }}\;\mathrm{dLo}g_{10}\left(\tau \right)}$$

This integration can be performed analytically for the cases *α* = 1 and *α* = 0 and numerically for 0 < *α* < 1.

The case *α* = 1: Here, all correlation times, homogeneously spaced in the logarithmic scale, are equally weighted. The expression for the relaxation rates becomes

(4) $$R_{1}=a+b\frac{\int_{\tau_{\min}}^{\tau_{\max}}\left[\frac{\tau }{1+\omega_{I}^{2}\tau^{2}}+\frac{4\tau }{1+4\omega_{I}^{2}\tau^{2}}\right]\;\mathrm{dLo}g_{10}\left(\tau \right)}{\int_{\tau_{\min}}^{\tau_{\max}}\mathrm{dLo}g_{10}\left(\tau \right)\;}$$

which is calculated equal to

(5) $$\begin{array}{c} R_{1}=a+\frac{b}{\mathrm{Lo}g_{10}\left(\tau_{\max}/\tau_{\min}\right)}\\ \frac{\left[\text{atan}\left(\omega_{I}\tau_{\max}\right)+2\text{atan}\left(2\omega_{I}\tau_{\max}\right)-\text{atan}\left(\omega_{I}\tau_{\min}\right)-2\text{atan}\left(2\omega_{I}\tau_{\min}\right)\right]}{\omega_{I}\ln\left(10\right)} \end{array}$$

For $\omega_{I}$ approaching zero, this expression approaches to the value $a+\frac{5b\left(\tau_{\max}-\tau_{\min}\right)}{\left[\mathrm{Lo}g_{10}\left(\tau_{\max}/\tau_{\min}\right)\right]\ln\left(10\right)}$. On the contrary, for values of $\omega_{I}$ > > 1/ $\tau_{\max}$ and << 1/ $\tau_{\min}$, $R_{1}$ can be approximated by $a+\frac{b}{\mathrm{Lo}g_{10}\left(\tau_{\max}/\tau_{\min}\right)}\frac{\left[3\frac{\pi }{2}\right]}{\omega_{I}\ln\left(10\right)}$.

This shows that, in this frequency range, the relaxation rates decay with the power‐law function 1/ $\omega_{I}$. Finally, for $\omega_{I}$ >> 1/ $\tau_{\min}$, $R_{1}$ decays to zero following the power‐law function 1/ $\omega_{I}^{2}$.

The case *α =* 0: Here, the expression for the relaxation rates becomes

(6) $$R_{1}=a+b\frac{\int_{\tau_{\min}}^{\tau_{\max}}\left[\frac{1}{1+\omega_{I}^{2}\tau^{2}}+\frac{4}{1+4\omega_{I}^{2}\tau^{2}}\right]\;\mathrm{dLo}g_{10}\left(\tau \right)}{\int_{\tau_{\min}}^{\tau_{\max}}\frac{1}{\tau }\mathrm{dLo}g_{10}\left(\tau \right)\;}$$

which is calculated equal to

(7) $$\begin{array}{c} R_{1}=a+\frac{b}{\frac{1}{\tau_{\min}}-\frac{1}{\tau_{\max}}}\\ \left[\ln\frac{\tau_{\max}}{\sqrt{1+\omega_{I}^{2}\tau_{\max}^{2}}}+4\ln\frac{\tau_{\max}}{\sqrt{1+4\omega_{I}^{2}\tau_{\max}^{2}}}-\ln\frac{\tau_{\min}}{\sqrt{1+\omega_{I}^{2}\tau_{\min}^{2}}}-4\ln\frac{\tau_{\min}}{\sqrt{1+4\omega_{I}^{2}\tau_{\min}^{2}}}\right] \end{array}$$

In this case, the frequency dependence of the relaxation rates cannot be described by a power‐law function.

The case 0 < *α* < 1: In the case of *α* between 0 and 1, as also previously observed [8, 9], the integral at the numerator of Equation (3) can be expressed analytically only using the Gaussian hypergeometric function, denoted as $F_{1}^{2}\left(1,\frac{\alpha }{2};1+\frac{\alpha }{2};-\omega_{I}^{2}\tau^{2}\right)$, a special function that can be calculated using various series expansions. It is thus more convenient to compute the expression numerically, by sampling *N* + 1 correlation times $\tau_{n}$ spaced logarithmically (i.e., at uniform intervals in ${\mathrm{Log}}_{10}\left(\tau \right)$). In this case, Equation (3) can be written as

(8) $$\begin{array}{c} R_{1}=a+b\frac{\ln\left(10\right)\left(\alpha -1\right)}{\tau_{\max}^{\alpha -1}-\tau_{\min}^{\alpha -1}}\frac{{\mathrm{Log}}_{10}\left(\tau_{\max}/\tau_{\min}\right)}{N}\\ \;\sum_{n=0}^{N}k_{n}\left[\frac{\tau_{n}^{\alpha }}{1+\omega_{I}^{2}\tau_{n}^{2}}+\frac{4\tau_{n}^{\alpha }}{1+4\omega_{I}^{2}\tau_{n}^{2}}\right] \end{array}$$

where $k_{n}=1$ for n≠0,N, $k_{0}=k_{N}=0.5$, and $\tau_{n}=10^{{\mathrm{Log}}_{10}\left(\tau_{\min}\right)+\frac{n}{N}\left[{\mathrm{Log}}_{10}\left(\tau_{\max}/\tau_{\min}\right)\right]}$.

When *α* = 1, Equation (8) can be replaced by

(9) $$R_{1}=a+\frac{b}{N}\sum_{n=0}^{N}k_{n}\left[\frac{\tau_{n}}{1+\omega_{I}^{2}\tau_{n}^{2}}+\frac{4\tau_{n}}{1+4\omega_{I}^{2}\tau_{n}^{2}}\right]$$

### 3.1 Simulated Profiles

**FIGURE 1:**
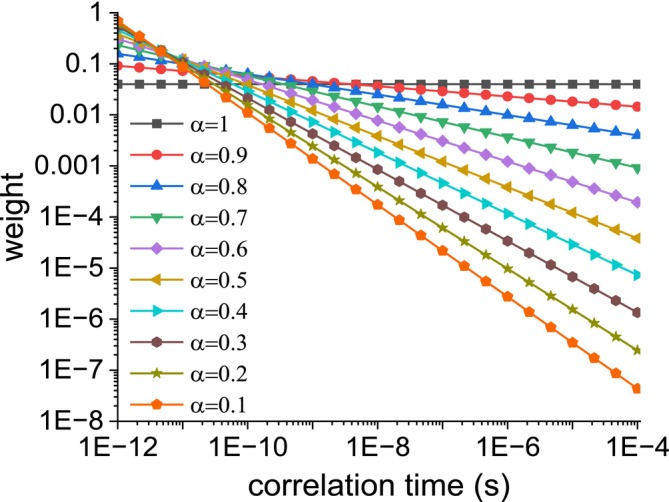
Weights of the spectral density functions for the different values of the correlation times used in the simulation of the relaxation rates reported in Figure 2.

**FIGURE 2:**
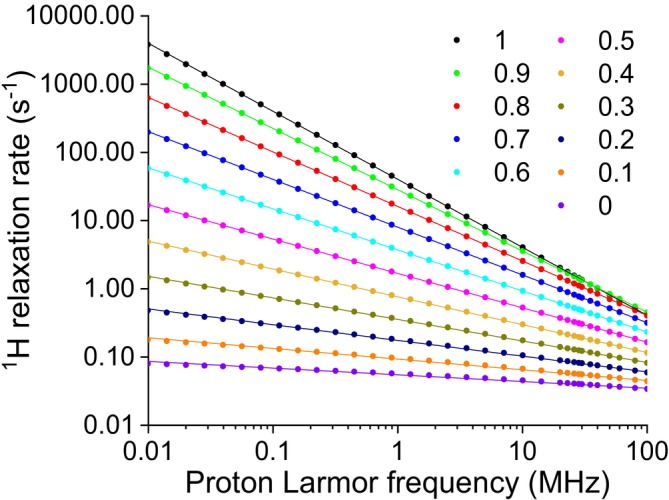
Simulated ^1^H NMRD data calculated using an inverse‐scaling distribution of correlation times from *τ*_min_ = 10^−12^ s to *τ*_max_ = 10^−4^ s (symbols) with weight $\frac{1}{\tau^{1-\alpha }}$, *α* varying from 0 to 1 in steps of 0.1, and best fit profiles (solid lines) obtained with a power‐law function. The best fit parameters are reported in Table 1.

^1^H NMRD profiles were simulated to reproduce the relaxation rates due to proton–proton dipole–dipole interactions modulated by stochastic fluctuations with a distribution of correlation times with weights 1/*τ* ^(1‐*α*)^ (inverse‐scaling distribution), for 0 < *α <* 1, in steps of 0.1, with *τ*_min_ = 10^−12^ s and *τ*_max_ = 10^−4^ s. The resulting weights of the spectral density functions for the different correlation times are reported in Figure 1. The relaxation data, shown in Figure 2 as solid circles, were calculated from 0.01 to 100 MHz ^1^H Larmor frequency, using Equation (8) (or Equation 9 for *α* = 1) and *N* = 24. These data are in excellent agreement with the profiles obtained with Equations (5) and (7) for the cases *α* = 1 and 0, respectively. In the simulation, the following values for the parameters were used: *a* = 0, *b* = 10^9^ s^−2^.

The simulated profiles clearly show a linear dependence in the log–log scale, so that they could be well fitted to the power‐law function.

(10) $$R_{1}=A\omega_{I}^{-\beta }$$

The best fit profiles are shown as solid lines in Figure 2, and the best fit parameters are reported in Table 1. The analysis shows that indeed the value of the parameter β is in good agreement with the weighing parameter *α* for *α* > 0.1 and in excellent agreement for 0.4 ≤ *α* ≤ 1.

**TABLE 1:** Best fit values of the simulated profiles shown in Figure 2.

*α*	*A* (s^−1−*β*^)	β
1	(2.40 ± 0.05) × 10^8^	0.997 ± 0.001
0.9	(3.60 ± 0.07) × 10^7^	0.898 ± 0.001
0.8	(4.35 ± 0.09) × 10^6^	0.799 ± 0.001
0.7	(4.60 ± 0.09) × 10^5^	0.700 ± 0.001
0.6	(4.56 ± 0.09) × 10^4^	0.601 ± 0.001
0.5	(4.46 ± 0.09) × 10^3^	0.504 ± 0.001
0.4	452 ± 9	0.408 ± 0.001
0.3	50.0 ± 1.0	0.316 ± 0.001
0.2	6.48 ± 0.13	0.231 ± 0.001
0.1	1.08 ± 0.02	0.157 ± 0.001
0	0.256 ± 0.005	0.098 ± 0.01

**FIGURE 3:**
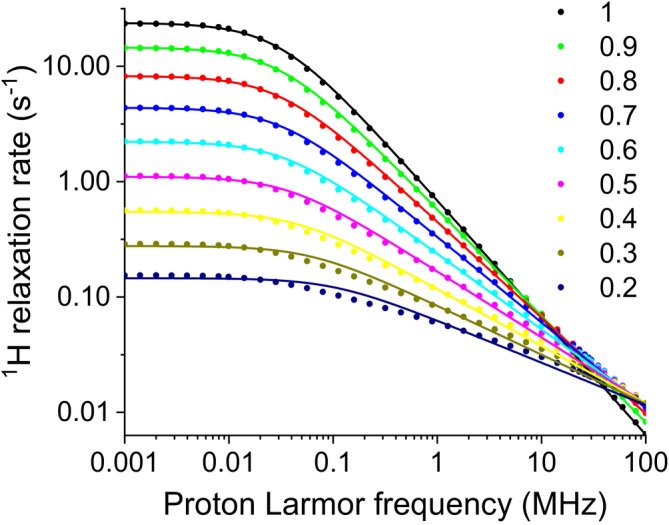
Simulated ^1^H NMRD data calculated using an inverse‐scaling distribution of correlation times from *τ*_min_ = 10^−10^ s to *τ*_max_ = 5 × 10^−6^ s (symbols) with weight $\frac{1}{\tau^{1-\alpha }}$, *α* varying from 0.2 to 1, and best fit profiles (solid lines) obtained with the power‐law function in Equation (11). The best fit parameters are reported in Table 2.

^1^H NMRD profiles were also simulated using Equation (8) (or Equation 9 for *α* = 1) with a distribution of correlation times from *τ*_min_ = 10^−10^ s to *τ*_max_ = 5 × 10^−6^ s and with *a* = 0, *b* = 10^7^ s^−2^. The relaxation data, shown in Figure 3 as solid circles, were calculated from 0.001 to 100 MHz ^1^Η Larmor frequency. Again, the data are in excellent agreement with the profile obtained with Equation (5) in the case *α* = 1.

Crucially, the power‐law regime does not extend indefinitely, as it also happens in many experimental cases: It only holds above a certain frequency. Phenomenological power‐law models are in fact typically valid only over a limited range of frequencies. In these cases, the simulated profiles can be fitted to the power‐law function.

(11) $$R_{1}=A{\left(\omega_{I}^{1.4}+\omega_{0}^{1.4}\right)}^{-\beta /1.4}$$

where the term $\omega_{0}$ is included to reproduce the flattening of the profiles toward constant values as frequencies approach zero. The exponent of 1.4 appearing in Equation (11) was determined empirically to achieve the best fit for the data with *α* = 1. The best fit profiles are shown as solid lines in Figure 3, and the best fit parameters are reported in Table 2. The analysis shows that, indeed, the value of the parameter β is in good agreement with the weighing parameter *α* for *α* > 0.6 and anyway decreases with *α* for *α* > 0.2. Thus, the value of $\omega_{0}$ provides a good estimate of the maximum correlation time, being

(12) $$\tau_{\max}\approx \omega_{0}^{-1}$$

**TABLE 2:** Best fit values of the simulated profiles shown in Figure 3.

	*A* (s^−1−*β*^)	β	$\omega_{0}^{-1}$ (s)
*α* = 1	(5.5 ± 0.7) × 10^6^	1.02 ± 0.01	(5.2 ± 0.3) × 10^−6^
*α* = 0.9	(1.1 ± 0.1) × 10^6^	0.92 ± 0.01	(5.3 ± 0.3) × 10^−6^
*α* = 0.8	(2.1 ± 0.3) × 10^5^	0.83 ± 0.01	(5.3 ± 0.4) × 10^−6^
*α* = 0.7	(3.7 ± 0.5) × 10^4^	0.74 ± 0.01	(5.1 ± 0.4) × 10^−6^
*α* = 0.6	(6.9 ± 0.9) × 10^3^	0.66 ± 0.01	(4.8 ± 0.4) × 10^−6^
*α* = 0.5	(1.3 ± 0.2) × 10^3^	0.57 ± 0.01	(4.3 ± 0.5) × 10^−6^
*α* = 0.4	274 ± 37	0.50 ± 0.01	(3.6 ± 0.4) × 10^−6^
*α* = 0.3	66 ± 10	0.43 ± 0.01	(2.6 ± 0.4) × 10^−6^
*α* = 0.2	20 ± 3	0.37 ± 0.01	(1.6 ± 0.3) × 10^−6^

This estimate is indeed excellent for *α* > 0.5.

### 3.2 Experimental Data

#### 3.2.1 Sedimented BSA

^1^H NMRD profiles of dry or lowly hydrated proteins have often been fitted using the power‐law model [1, 21, 22, 23, 24]. The ^1^H NMRD profile of sedimented bovine serum albumin (BSA) [23], for instance, can be fitted with a power law model that sums up to an additional contribution arising from a dipolar interaction modulated from a single correlation time, plus a constant term *a*:

(13) $$R_{1}=A{\left(\omega_{I}^{1.4}+\omega_{0}^{1.4}\right)}^{-\beta /1.4}+b\left[\frac{\tau_{c}}{1+\omega_{I}^{2}\tau_{c}^{2}}+\frac{4\tau_{c}}{1+4\omega_{I}^{2}\tau_{c}^{2}}\right]+a$$

**FIGURE 4:**
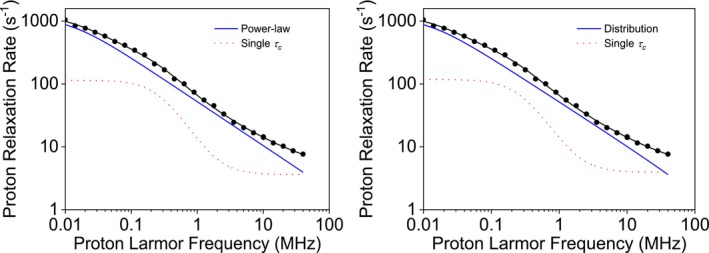
Experimental ^1^H NMRD profiles of sedimented BSA [23], best fit profiles, and their contributions, obtained from the power‐law model (left panel) or from an inverse‐scaling distribution of correlation times (right panel), in both cases including the additional contribution from a single correlation time and a constant term.

**FIGURE 5:**
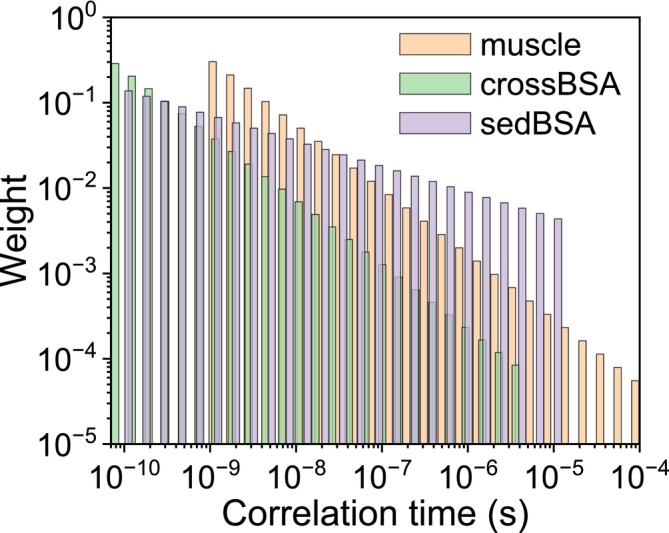
Distribution of correlation times used to reproduce the experimental NMRD profiles of sedimented BSA, muscle tissue, and cross‐linked BSA.

Six parameters are used in the fit, that is, *a*, *b*, $\tau_{c}$, *A*, β, and $\omega_{0}$. An equally excellent fit is obtained using a sum of Lorentzian functions with an inverse‐scaling distribution of correlation times (Equation 3) and an additional contribution arising from a dipolar interaction modulated from a single correlation time, plus a constant term:

(14) $$\begin{array}{c} R_{1}=b_{D}\frac{\ln\left(10\right)\left(\alpha -1\right)}{\tau_{\max}^{\alpha -1}-\tau_{\min}^{\alpha -1}}\frac{{\mathrm{Log}}_{10}\left(\tau_{\max}/\tau_{\min}\right)}{N}\sum_{n=0}^{N}k_{n}\left[\frac{\tau_{n}^{\alpha }}{1+\omega_{I}^{2}\tau_{n}^{2}}+\frac{4\tau_{n}^{\alpha }}{1+4\omega_{I}^{2}\tau_{n}^{2}}\right]\\ +b\left[\frac{\tau_{c}}{1+\omega_{I}^{2}\tau_{c}^{2}}+\frac{4\tau_{c}}{1+4\omega_{I}^{2}\tau_{c}^{2}}\right]+a \end{array}$$

where $k_{n}=1$ for n≠0,N, $k_{0}=k_{N}=0.5$, $\tau_{n}=10^{{\mathrm{Log}}_{10}\left(\tau_{\min}\right)+\frac{n}{N}\left[{\mathrm{Log}}_{10}\left(\tau_{\max}/\tau_{\min}\right)\right]}$, and *N* = 24. Also in this model, six parameters have been fitted, that is, *a*, *b*, $\tau_{c}$, $b_{D}$, α, and $\tau_{\max}$ (the value of $\tau_{\min}$ was fixed to 10^−10^ s). The best fit profiles obtained with the two models and their contributions are shown in Figure 4, and the resulting best fit parameters are reported in Table 3. Notably, the best fit values of α and β coincide, the values of $\tau_{c}$ are the same within the error, and $\omega_{0}^{-1}$ is in good agreement with $\tau_{\max}$. The distribution of correlation times obtained from the fit is shown in Figure 5. It spans from 10^−10^ to 10^−5^ s, with a weighted mean of 1.6 × 10^−7^ s, much higher than the most probable correlation times (equal to 10^−10^ s). This indicates that although the short timescales hold the majority of the weight, a long tail of correlation times extending toward larger values, despite their small individual weights, largely influences the overall mean. This wide spread of correlation times also reflects in a standard deviation of 9.0 × 10^−7^ s, five to six times larger than the weighted mean.

**TABLE 3:** Best fit parameters obtained from the NMRD profiles of sedimented BSA using either the power‐law model or a sum of Lorentzian functions with an inverse‐scaling distribution of correlation times.

	Power law	Inverse‐scaling distribution
*a* (s^−1^)	3.6 ± 0.5	3.9 ± 0.5
*b* (s^−2^)	(7.1 ± 1.2) × 10^7^	(7.2 ± 1.1) × 10^7^
*τ*_*c*_ (s)	(3.1 ± 0.4) × 10^−7^	(3.2 ± 0.4) × 10^−7^
*A* (s^−1−*β*^)	(3.0 ± 1.4) × 10^6^	
β	0.70 ± 0.03	
$\omega_{0}^{-1}$ (s)	(1.4 ± 0.4) × 10^−5^	
*b*_*D*_ (s^−2^)		(1.6 ± 0.1) × 10^9^
*α*		0.70 ± 0.03
*τ*_max_ (s)		(1.0 ± 0.3) × 10^−5^
*τ*_min_ (s)		10^−10^ (fixed)

#### 3.2.2 Muscle Tissue

**FIGURE 6:**
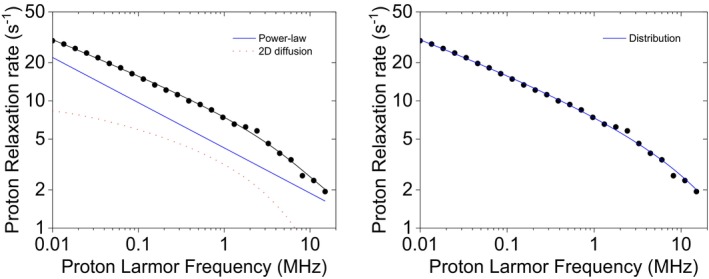
Experimental ^1^H NMRD profiles of mouse leg tissue [25], best fit profiles, and their contributions, obtained from the power‐law model and 2D translational diffusion (left panel) or from an inverse‐scaling distribution of correlation times (right panel).

Excised biological tissues often necessitate the use of multicomponent models including the power‐law term, associated with the interaction between water molecules and long‐lived protein binding sites, and a logarithmic term, accounting for the 2D translational dynamics of water at macromolecular interfacial regions. For instance, the ^1^H NMRD profiles of a muscle tissue [25], shown in Figure 6, can be fitted using the power‐law model that sums up to contributions from the 2D translational diffusion model, plus a constant term:

(15) $$R_{1}=A{\left(\omega_{I}^{1.4}+\omega_{0}^{1.4}\right)}^{-\beta /1.4}+\beta_{T}\tau_{D}\left[\ln\frac{1+\omega^{2}\tau_{D}^{2}}{\frac{\tau_{D}^{2}}{\tau_{\mathrm{res}}^{2}}+\omega^{2}\tau_{D}^{2}}+4\ln\frac{1+4\omega^{2}\tau_{D}^{2}}{\frac{\tau_{D}^{2}}{\tau_{\mathrm{res}}^{2}}+4\omega^{2}\tau_{D}^{2}}\right]+a$$

where $\tau_{D}$ is the correlation time for the two‐dimensional translational diffusion of the water molecules on the macromolecule surface and *τ*_res_ is the lifetime of the water molecules undergoing two‐dimensional translation diffusion in the vicinity of the surface of the macromolecule. Four parameters are used in the fit, that is, *A*, β, $\beta_{T}$, $\tau_{D}$ (*τ*_res_, usually much longer than $\tau_{D}$, was fixed to 10^−5^ s, $\omega_{0}$ was fixed to 0, and *a* was fixed to 0). Again, an equally excellent fit is obtained using Equation (14) and three fit parameters: $b_{D}$, α, and $\tau_{\min}$ ( $\tau_{\max}$ was fixed to 10^−4^ s, and *a* and *b* were fixed to 0). The best fit profiles obtained with the two models and their contributions are shown in Figure 6, and the resulting best fit parameters are reported in Table 4. Remarkably, the inverse‐scaling distribution model does not require additional contributions to fit the data. In this case, the obtained value of *α* is somewhat smaller than *β*, in agreement with the simulation performed for low *α* values. The distribution of correlation times obtained from the fit is shown in Figure 5.

**TABLE 4:** Best fit parameters obtained from the NMRD profiles of mouse leg tissue using either the power‐law model or a sum of Lorentzian functions with an inverse‐scaling distribution of correlation times.

	Power law	Inverse‐scaling distribution
*a* (s^−1^)	0	0
*A* (s^−1−*β*^)	(1.1 ± 0.3) × 10^3^	
β	0.36 ± 0.03	
$\omega_{0}$ (s)	0	
$\beta_{T}$ (s^−2^)	(4.1 ± 2.3) × 10^7^	
$\tau_{D}$ (s)	(6.8 ± 3.0) × 10^−9^	
*τ*_res_ (s)	10^−5^ (fixed)	
*b*_*D*_ (s^−2^)		(2.0 ± 0.3) × 10^8^
*α*		0.24 ± 0.01
*τ*_max_ (s)		10^−4^ (fixed)
*τ*_min_ (s)		(1.2 ± 0.3) × 10^−9^

#### 3.2.3 Cross‐Linked BSA

**FIGURE 7:**
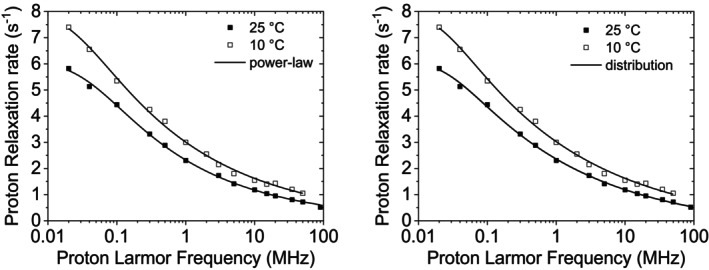
Experimental ^1^H NMRD profiles of 10% w/w cross‐linked BSA at 10°C and 25°C [26] and best fit profiles obtained from the power‐law model (left panel) or from an inverse‐scaling distribution of correlation times (right panel).

^1^H NMRD profiles of 10% w/w cross‐linked BSA at 25°C and 10°C [26] are shown in Figure 7. The profiles can be fitted equally well using the power‐law model, with fitting parameters *A*, β, and $\omega_{0}^{-1}$, or with an inverse‐scaling distribution of correlation times (Equation 3), with fitting parameters *b*_*D*_, *α*, *τ*_min_, *τ*_max_, and *a*, with common values for *b*_*D*_ and *α* (on the contrary, the values of β cannot be the same for both temperatures). Again, the best fit values of α and β are in relatively good agreement as well as the values of $\omega_{0}^{-1}$ and $\tau_{\max}$ (Table 5). The distribution of correlation times obtained from the fit is shown in Figure 5.

**TABLE 5:** Best fit parameters obtained from the NMRD profiles of cross‐linked BSA using either the power‐law model or a sum of Lorentzian functions with an inverse‐scaling distribution of correlation times.

	Power law		Inverse‐scaling distribution	
	25°C	10°C	25°C	10°C
*a* (s^−1^)	0	0	0.13 ± 0.07	0.39 ± 0.09
*A* (s^−1−*β*^)	217 ± 16			
β	0.29 ± 0.01	0.27 ± 0.01		
$\omega_{0}^{-1}$ (s)	(4.8 ± 0.5) × 10^−6^	(5.9 ± 0.8) × 10^−6^		
*b*_*D*_ (s^−2^)			(3.3 ± 0.3) × 10^8^	
*α*			0.24 ± 0.02	
*τ*_max_ (s)			(3.6 ± 0.7) × 10^−6^	(4.6 ± 1.0) × 10^−6^
*τ*_min_ (s)			1.0 × 10^−10^	(1.3 ± 0.1) × 10^−10^

#### 3.2.4 Hyaluronic Acid

**FIGURE 8:**
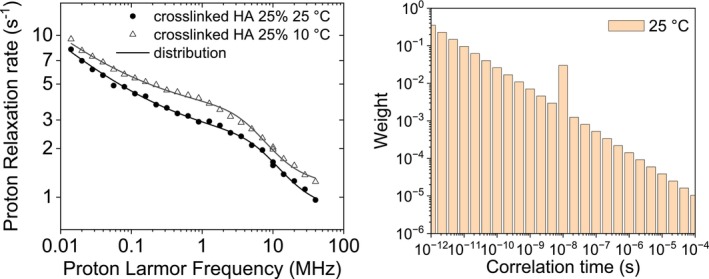
Experimental ^1^H NMRD profiles of crosslinked HA (with concentration of the HA polymers of 25% by weight) at 25°C and 10°C [31]. The lines are the best fit profiles calculated using a distribution of correlation time and an additional contribution from a single correlation time (right panel).

Hyaluronic acid (HA) is a natural component of skin and many connective tissues, an important component of the extracellular matrix, and it is used as a viscoelastic biomaterial for medical purposes [27, 28]. HA hydrogels have been prepared by crosslinking HA polymers and were shown useful for regenerating functional tissues [29]. Crosslinked HA hydrogels have high water content and biophysical and biochemical properties similar to many biological tissues [30]. Samples of highly concentrated crosslinked HA were prepared by lyophilizing the commercial Hylastan SGL‐80 sample and then rehydrating it to smaller extents [31]. The relaxation profiles of crosslinked HA for a concentration of the HA polymer corresponding to 25% by weight of the polysaccharide were collected at 10°C and 25°C [31] (Figure 8). The strong field dependence of the relaxation rates indicates the presence of dynamics on multiple timescales. These profiles could be satisfactorily fitted using three correlation times (equal to 1.5 × 10^−5^, 5.9 × 10^−7^ and 1.8 × 10^−8^ s, at 25°C), corresponding to three different pools of water molecules with different dynamics. The profiles can also be fitted using Equation (14), which comprises a sum of Lorentzian functions with an inverse‐scaling distribution of correlation times and an additional contribution arising from a dipolar interaction modulated from a single correlation time, plus a constant term. The best profiles are shown in Figure 8, and the fit values are *a*: 0.73 ± 0.05 and 1.08 ± 0.05 s^−1^, at 25°C and 10°C, respectively; *b*: (2.6 ± 0.1) × 10^7^ s^−2^; *τ*_*c*_: (1.0 ± 0.1) × 10^−8^ and (1.5 ± 0.1) × 10^−8^ s, at 25°C and 10°C, respectively; *b*_*D*_: (9.2 ± 3.1) × 10^8^ s^−2^; *α*: 0.43 ± 0.03; *τ*_max_: fixed to 10^−4^ s; and *τ*_min_: fixed to 10^−12^ s.

#### 3.2.5 Asparaginase

**FIGURE 9:**
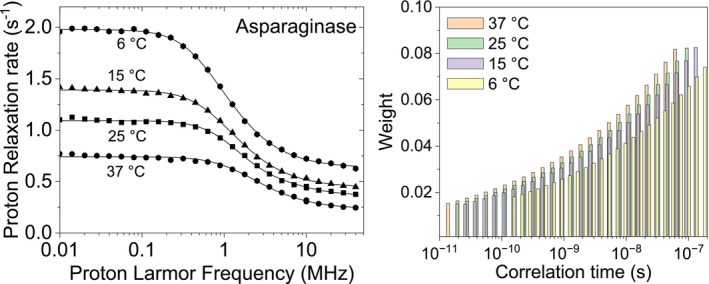
Experimental ^1^H NMRD profiles of L‐asparaginase II (0.8 mM) at different temperatures (left panel) [32, 33]. The lines are the best fit profiles calculated with the distribution of correlation times shown in the right panel.

Water molecules forming the hydration shell around proteins in diluted solution can exist in a highly heterogeneous environment, leading to dynamics that cannot be represented by the single correlation time corresponding to a rigid reorientation by macromolecular tumbling. For this reason, a distribution of correlation times has been used to fit the relaxation profiles of L‐asparaginase II (0.8 mM) collected at different temperatures [32, 33]. This is a diamagnetic protein composed of four identical subunits, forming a dimer of dimers of 138 kDa. The reorientation correlation time is thus expected to be of the order of 50 ns at 25°C. However, this correlation time cannot provide alone a good fit of the data and either three correlation times or addition of 2D translational diffusion (second term in Equation 15) should be considered to reproduce the data [2]. The profiles can also be fitted using an inverse‐scaling distribution of correlation times (Figure 9). The resulting best fit parameters are reported in Table 6. Differently from the previous cases, the parameter *α* is larger than 1, so that the weights increase with increasing the correlation times. At 25°C, the weighted average of the correlation times is 2.4 × 10^−8^ s, with a standard deviation of 3.1 × 10^−8^ s.

**TABLE 6:** Best fit parameters obtained from the NMRD profiles of L‐asparaginase II (0.8 mM) using an inverse‐scaling distribution of correlation times.

	37°C	25°C	15°C	6°C
*a* (s^−1^)	0.23 ± 0.01	0.36 ± 0.01	0.44 ± 0.01	0.63 ± 0.01
*b*_*D*_ (s^−2^)	(7.3 ± 0.8) × 10^6^			
*α*	1.20 ± 0.06			
*τ*_max_ (s)	(6.7 ± 0.3) × 10^−8^	(9.7 ± 0.4) × 10^−8^	(1.3 ± 0.1) × 10^−7^	(1.7 ± 0.1) × 10^−7^
*τ*_min_ (s)	(1.5 ± 1.5) × 10^−11^	2.0 × 10^−11^ (fixed)	(2.5 ± 2.0) × 10^−11^	(1.4 ± 0.9) × 10^−10^

#### 3.2.6 Double‐Scaling Distribution

**FIGURE 10:**
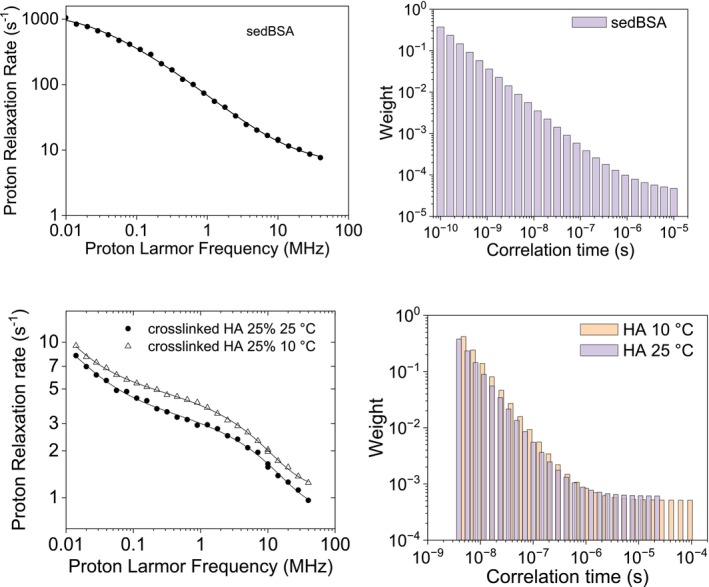
Experimental ^1^H NMRD profiles of sedimented BSA and crosslinked hyaluronic acid (left panels). The lines are the best fit profiles calculated with the double‐scaling distribution of correlation times shown in the right panels.

All profiles showed above could be well fitted with the modeled inverse‐scaling distribution of correlation times except the cases of sedimented BSA and crosslinked hyaluronic acid, where an additional contribution arising from a single correlation time was needed. In these cases, a new model for the distribution of the correlation times can be introduced, allowing for a change in the scaling exponent on passing from fast to slow correlation times. A double‐scaling distribution was thus modeled with weights.

(16) $$w\left(\tau_{n}\right)={\left(\frac{\tau_{\text{node}}}{\tau_{n}}\right)}^{1-\alpha }{\left(1+\frac{\tau_{n}}{\tau_{\text{node}}}\right)}^{\gamma +1-\alpha }$$

where *τ*_node_ indicates the correlation time at which the change occurs. Using this double‐scaling distribution of correlation times, excellent fits can be obtained both for sedimented BSA and crosslinked hyaluronic acid (see Figure 10 and Table 7). Some parameters should be held fixed in the fit due to their large covariances. The distribution of correlation times obtained from the fit is also shown in Figure 10.

**TABLE 7:** Best fit parameters obtained from the NMRD profiles of sedimented BSA and crosslinked hyaluronic acid using a double‐scaling distribution of correlation times.

	Sedimented BSA	Crosslinked HA	
		25°C	10°C
*a* (s^−1^)	5.9 ± 0.04	0.81 ± 0.03	1.13 ± 0.03
*b*_*D*_ (s^−2^)	(9.9 ± 0.7) × 10^8^	(4.6 ± 0.3) × 10^7^	
*α*	0.96 ± 0.03	−0.34 ± 0.07	
*γ*	−0.98 ± 0.08	0 (fixed)	
*τ*_max_ (s)	10^–5^ (fixed)	(2.1 ± 1.0) × 10^−5^	10^–4^ (fixed)
*τ*_min_ (s)	10^–10^ (fixed)	(3.7 ± 0.4) × 10^−9^	(5.1 ± 0.5) × 10^−9^
*τ*_node_ (s)	10^–6^ (fixed)	(4.5 ± 1.0) × 10^−7^	(7.6 ± 1.5) × 10^−7^

## 4 Conclusion

Power‐law frequency dependences represent a crucial characteristic of NMRD profiles of systems governed by slow and highly constrained molecular motions present across various macromolecular solutions, tissues, and hydrogels. Based on the extreme‐values statistics of rare events leading to the Pareto distribution of correlation times, the power‐law exponent can provide a quantitative marker of underlying molecular dynamics, such as polymer chain dynamics or water diffusion in constrained environments. Numerical simulations and experimental analyses across crosslinked proteins, tissues, hydrogels, and polymer networks show that the power‐law exponent corresponds closely to the weighting parameter governing the distribution of correlation times. The distribution‐based approach reproduces experimental data with accuracy equal to or exceeding that of phenomenological power‐law fits, but with the advantage of directly yielding the underlying timescale distribution. In some experimental cases (such as sedimented proteins and cross‐linked hyaluronic acid), a single inverse‐scaling distribution is insufficient to reproduce the full shape of the relaxation profiles. For these systems, a double‐scaling distribution, featuring a crossover between fast and slow correlation‐time regimes, is able to reproduce the data accurately without the need for additional contributions. In summary, this distribution‐based approach enables the extraction of dynamic information inaccessible through empirical modeling through power‐law fits.

## Funding

This work was supported by the European Commission HORIZON MSCA‐DN FC‐RELAX (101072758) and the Fondazione Cassa di Risparmio di Firenze.

## Conflicts of Interest

The authors declare no conflicts of interest.

## Data Availability

Data sharing is not applicable to this article, as no datasets were generated or analyzed during the current study.
